# Assessment of retinal nerve fiber thickness and optic nerve head blood flow in female patients diagnosed with fibromyalgia syndrome

**DOI:** 10.5935/0004-2749.20220005

**Published:** 2022

**Authors:** Selma Urfalıoglu, Ejder Berk

**Affiliations:** 1 Department of Ophthalmology, Faculty of Medicine, Kahramanmaras Sutcu Imam University, Kahramanmaras, Turkey; 2 Department of Physical medicine and Rehabilitation, Faculty of Medicine, Kahramanmaras Sutcu Imam University, Kahramanmaras, Turkey

**Keywords:** Fibromyalgia, Tomography, optical coherence, Angiography, Optic nerve/blood supply, Nerve fibers, Retina, Fibromialgia, Tomografia de coerência óptica, An giografia, Nervo óptico/irrigação sanguínea, Fibras nervosas, Retina

## Abstract

**Purpose:**

Numerous neuroimaging and ophthalmic studies suggest optic nerve involvement in fi bromyalgia syndrome. To further elucidate the etiopathogenesis of fibromyalgia syndrome, we compared optic nerve head blood flow area and retinal nerve fiber layer thickness between patients and controls and investigated the associations of these measures with fibromyalgia syndrome severity.

**Methods:**

Participants were divided into the following three groups according to Fibromyalgia Impact Questionnaire score: mild-moderate fibromyalgia syndrome (Group 1, n=47), severe fibromyalgia syndrome (Group 2, n=38), and healthy controls (Group 3, n=38). The optic nerve head blood flow area and retinal nerve fiber layer thickness were measured by optical coherence tomography angiography and compared among groups by ANOVA. Associations with fibromyalgia syndrome severity were evaluated by Spearman’s correlation analysis.

**Results:**

Optic nerve head blood flow area did not differ between fibromyalgia syndrome Groups 1 and 2 (1.61 ± 0.08 vs.1.63 ± 0.09 mm^2^), but it was significantly lower in control Group 3 (1.49 ± 0.10 mm^2^, all p=0.001). Average retinal nerve fiber layer thickness values were significantly lower in Group 2 (101.18 ± 6.03 µm) than in Group 1 (103.21 ± 10.66 µm) and Group 3 (106.51 ± 8.88 µm) (p=0.041 and 0.020, respectively). The inferotemporal (134.36 ± 12.19 µm) and inferonasal (109.47 ± 16.03 µm) quadrant retinal nerve fiber layer thickness values were significantly lower in Group 2 than in Group 1 [inferotemporal (142.15 ± 17.79 µm), inferonasal (117.94 ± 20.53 µm)] and Group 3 [inferotemporal (144.70 ± 16.25 µm), inferonasal (118.63 ± 19.01 µm)] [inferotemporal, p=0.017 and 0.010, respectively; inferonasal, p=0.047 and 0.045, respectively]. The nasal-superior quadrant retinal nerve fiber layer thickness value was higher in Group 3 (91.08 ± 12.11 µm) than in Group 1 (84.34 ± 13.09 µm) and Group 2 (85.26 ± 13.11 µm) (p=0.031 and 0.038, respectively). A weak correlation was detected between disease severity and optic nerve head blood flow area.

**Conclusion:**

Neural and vascular structures of the eye are altered in fibromyalgia syndrome, particularly among severe cases. Therefore, optical coherence tomography angiography may provide valuable information for the diagnosis and elucidation of fibromyalgia syndrome pathophysiology.

## Abstract (pt)

**Objetivo:**

Numerosos estudos de neuroimagem e oftalmologia sugerem o acometimento do nervo óptico na síndrome de fibromialgia. Para esclarecer a etiopatogenia da síndrome de fibromialgia, comparamos a área de fluxo sanguíneo da cabeça do nervo óptico e a espessura da camada de fibras nervosas da retina entre pacientes e o grupo controle, e examinamos as associações dessas medidas com a severidade da síndrome de fibromialgia.

**Métodos:**

Os participantes foram divididos em três grupos de acordo com a pontuação no Questionário de Impacto da Fibromialgia: síndrome de fibromialgia leve a moderada (Grupo 1, n=47), síndrome de fibromialgia grave (Grupo 2, n=38) e controles saudáveis (Grupo 3, n=38). A área de fluxo sanguíneo da cabeça do nervo óptico e a espessura da camada de fibras nervosas da retina foram medidas por angiotomografia de coerência óptica e comparadas entre os grupos por ANOVA. As associações com a severidade da síndrome de fibromialgia foram avaliadas pela análise de correlação de Spearman. **Resultados**:

A área de fluxo sanguíneo da cabeça do nervo óptico não diferiu entre os Grupos 1 e 2 da síndrome de fibromialgia (1,61 ± 0,08 contra 1,63 ± 0,09 mm^2^), mas foi significativamente menor no Grupo 3, de controle (1,49 ± 0,10 mm^2^, todos com p=0,001). Os valores médios da espessura da camada de fibras nervosas da retina foram significativamente menores no Grupo 2 (101,18 ± 6,03 µm) em comparação com o Grupo 1 (103,21 ± 10,66 µm) e o Grupo 3 (106,51 ± 8,88 µm) (p=0,041, p=0,020). Os valores da espessura da camada de fibras nervosas no quadrante inferotemporal (134,36 ± 12,19 µm) e inferonasal (109,47 ± 16,03 µm) foram significativamente menores no Grupo 2 em comparação com o Grupo 1 (inferotemporal 142,15 ± 17,79 µm, inferonasal 117,94 ± 20,53 µm) e o Grupo 3 (inferotemporal 144,70 ± 16,25 µm, inferonasal 118,63 ± 19,01 µm) Para o quadrante inferotemporal, foram obtidos p=0,017 e p=0,010 para o Grupo 2 em comparação respectivamente com os Grupos 1 e 3, e para o quadrante inferonasal, p=0,047 e p=0,045, respectivamente para os mesmos grupos. A espessura da camada de fibra nervosa da retina no quadrante nasal superior foi maior no Grupo 3 (91,08 ± 12,11 µm) que no Grupo 1 (84,34 ± 13,09 µm) e no Grupo 2 (85,26 ± 13,11 µm); p=0,031 e p=0,038. Houve uma correlação fraca entre a severidade da doença e a área de fluxo sanguíneo na cabeça do nervo óptico.

**Conclusão:**

As estruturas neurais e vasculares do olho estão alteradas na síndrome da fibromialgia, principalmente nos casos graves. Assim, a angiografia por tomografia de coerência óptica pode fornecer informações valiosas para o diagnóstico e elucidação da fisiopatologia da síndrome de fibromialgia.

## INTRODUCTION

Fibromyalgia syndrome (FMS) is a multifactorial disorder characterized by chronic pain, fatigue, asthenia, and sleeping disorder among other symptoms^(1)^. Although its prevalence varies between 1% and 4% in the general population, it exhibits a remarkably higher prevalence in the adult female population (2.5%-10.5%) than in the adult male population (up to 4%)^(2-4)^. Numerous factors are implicated in the occurrence and progression of FMS, including genetic, environmental, and nutritional factors, viral infections, stress, and neuroendocrine anomalies. The physiopathology still remains incompletely understood; however, it is believed that sympathetic dysfunction, neuroendocrine anomalies, and psychogenic factors contribute to FMS by altering pain perception and threshold in the muscle tissue^(5,6)^. Moreover, neuroimaging studies have detected hypoperfusion in the thalamus and caudate nucleus of patients with FMS^(7-9)^, suggesting optic nerve involvement.

In patients with other neurodegenerative disorders, morphometric and functional evaluations of the optic nerve provide important information regarding axonal degeneration as well as visual dysfunction^(10,11)^. For instance, reduced retinal nerve fiber layer (RNFL) integrity at the optic nerve head as measured by optical coherence tomography (OCT) is frequently considered in the diagnosis of neurodegenerative disease. In addition to structural OCT, optical coherence tomography angiography (OCTA) measures of erythrocyte flow in retinal and optic nerve vessels can be used as a diagnostic tool, especially in ophthalmic vascular diseases such as diabetic retinopathy, embolism, age-related macular degeneration, and glaucoma^(12)^.

This study was conducted to analyze the changes in ophthalmic vascular and axonal structures in patients with FMS by evaluating the optic nerve head blood flow area (ONH-BFA) and RNFL thickness using OCTA.

## METHODS

All procedures conducted in studies involving human participants were in accordance with the ethical standards of the institutional and/or national research committee and with the 1964 Declaration of Helsinki and its later amendments or comparable ethical standards. This study was approved by the Clinical Research Ethics Committee of the Kahramanmaras Sutcu Imam University Faculty of Medicine (approval number-date: 323-25/07/2018).

### Study design

This was a single-center prospective study conducted in Physical Medicine and Rehabilitation and Ophthalmology clinics of the Kahramanmaras Sutcu Imam University Faculty of Medicine between July 2018 and June 2019. Female patients aged 18-60 years admitted to the Physical Medicine and Rehabilitation outpatient clinic with complaints of chronic body pain, weakness, fatigue, stiffness, and/or sleep disorder and diagnosed with new primary FMS according to the American College of Rheumatology (ACR) 2010 criteria were included^(13)^. All male patients treated during the recruitment period and female patients with a previous history of FMS treatment or currently receiving any medical treatment for FMS were excluded. Furthermore, female patients diagnosed with secondary FMS, cognitive impairment, any eye disease (refractive error, cataract, glaucoma, retinal diseases, uveitis, iridocyclitis, etc.), and systemic diseases (heart disease, hypertension, rheumatoid arthritis, vasculitis, neurological diseases, etc.) and patients unable to complete questionnaires due to illiteracy were excluded. The control group (Group 3, n=38) consisted of healthy volunteer women of a similar age range and no previously diagnosed rheumatologic or eye diseases.

Clinical interviews, OCTA measurements, and other evaluations were performed by the same physical medicine and rehabilitation specialists and ophthalmologists. Pain was evaluated using a visual analog scale (VAS), with 0 indicating no pain and 10 indicating very severe pain. Moreover, the Fibromyalgia Impact Questionnaire (FIQ) was administered to evaluate the severity of the disease.

### Fibromyalgia impact questionnaire

The FIQ was originally developed by Burckhardt et al.^(14)^ to evaluate the effects of FMS on multiple life domains, and its validity and reliability have been confirmed^(15)^. The FIQ consists of 10 questions evaluating physical function, occupational status, depression, anxiety, sleeping, pain, rigidness, fatigue, and general well-being. Each question is scored between 0 and 10 for an overall FIQ score in the range of 0-100 points, with higher scores representing a greater adverse impact on the patient’s life. In the present study, a score of ≤70 was defined as mild-moderate FMS (Group 1, n=47), and a score >70 was defined as severe FMS (Group 2, n=38)^(16)^.

### Ophthalmological examinations

All patients were examined by slit lamp biomicroscopy, applanation tonometry, and fundus examination.

### Optical coherence tomography angiography

The ONH-BFA, average RNFL thickness, and RNFL thicknesses of superotemporal, superonasal, inferotemporal, inferonasal, nasal-superior, nasal-inferior, temporal-superior, and temporal-inferior quadrants were measured using the RTVue XR Avanti Spectral domain OCT AngioVue (Optuvue, Inc, Fremont, CA, USA), a system that can provide 70 000 scans per second using an 840-nm light source. All measures were obtained according to the recommendations of the manufacturer for optic nerve scan and image capture. Images with poor resolution, motion artifacts, and/or a signal strength index >50 were eliminated. All measurements were recorded and calculated automatically using the AngioAnalytics software. Measurements were performed for both eyes, but only the right eye values were included in the statistical analyses. Optic nerve head measurements were acquired sequentially under normal lighting conditions without dilatation of the pupil. The ONH-BFA surface venous flow field was 3.14 mm^2^ and was acquired using the device 6 × 6 mm measurement mode. The AngioAna lytics software can calculate parameters in a specified cube. The flow is defined as the area occupied by the veins in a 1-mm-diameter circle and is expressed in units of mm^2^.

### Statistical analysis

According to a power analysis based on a pilot study of five control and five patients with mild-moderate FMS, 37 subjects per group were required to detect a significant difference with 90% power at α=0.05. All statistical analyses were conducted using SPSS 17.0 (IBM Corporation, Armonk, New York, USA). Before comparisons, all data distributions were tested for normality using the Shapiro-Wilk test and for variance homogeneity using Levene’s test. Continuous variables are presented in tables as mean ± standard deviation (*std*) or median and range (maximum-minimum) according to the Shapiro-Wilk test results, whereas categorical variables are represented as number (n) and percentage (%). Multiple groups were compared by one-way ANOVA with post hoc Tukey *HSD* tests for pairwise comparisons. Associations between OCTA metrics and disease severity (FIQ scores) were investigated by Spearman’s *rho* correlation analysis. A p value <0.05 (two-tailed) was considered to be significant for all tests.

## RESULTS

The mean age values of the patients included in this study were 44.90 ± 10.86 years in Group 1, 45.56 ± 9.19 years in Group 2, and 44.42 ± 10.72 years in Group 3, with no statistical difference being found (p=0.635). There was also no difference among Groups 1, 2, and 3 in terms of intraocular pressure (IOP) values (14.06 ± 2.96, 14.36 ± 3.11, and 14.65 ± 1.94 mmHg, respectively; p=0.611). The VAS scores were different among all groups; however, the highest score was found in Group 2 (8.30 ± 1.26; p=0.001). Table 1 shows the general characteristics of the study patients.

**Table 1:** General characteristics of the patients with FMS

	Group 1 (Mild-moderate FMS) (n=47)	Group 2 (Severe FMS) (n=38)	Group 3 (Control group) (n=38)	p-value
Age (years)	44.9 ± 10.86	45.56 ± 9.19	44.42 ± 10.72	0.635
IOP (mmHg)	14.06 ± 2.96	14.36 ± 3.11	14.65 ± 1.94	0.611
VAS score	6.74 ± 1.75	8.30 ± 1.26	1.89 ± 0.92	0.001^*^

One-way ANOVA; Kruskal-Wallis H test.

Post hoc test: Tukey HSD.

* *p*≤0.05, The difference between the three groups was statistically significant. IOP= Intraocular pressure; VAS= Visual analog scale.

When the study groups were evaluated in terms of ONH-BFA values, no difference was found between Group 1 (1.61 ± 0.08 mm^2^) and Group 2 (1.63 ± 0.09 mm^2^); however, the flow area values were significantly low in Group 3 (1.49 ± 0.10 mm^2^) (all p=0.001).

Regarding the comparison of the average RNFL thickness values, it was observed that the values were significantly lower in Group 2 (101.18 ± 6.03 µm) than in Group 1 (103.21 ± 10.66 µm) and Group 3 (106.51 ± 8.88 µm) (p=0.041 and 0.020, respectively). When the RNFL thickness values were analyzed by quadrants, a difference was found in Group 2 in terms of inferotemporal (134.36 ± 12.19 µm) and inferonasal (109.47 ± 16.03 µm) quadrant RNFL thickness values compared to those in Group 1 [inferotemporal (142.15 ± 17.79 µm), inferonasal (117.94 ± 20.53 µm)] and Group 3 [inferotemporal (144.70 ± 16.25 µm), inferonasal (118.63 ± 19.01 µm)] [inferotemporal, p=0.017 and 0.010, respectively; inferonasal, p=0.047 and 0.045, respectively]. Moreover, the nasal-superior quadrant RNFL thickness value was significantly higher in Group 3 (91.08 ± 12.11 µm) than in Group 1 (84.34 ± 13.09 µm) and Group 2 (85.26 ± 13.11 µm) (p=0.031 and 0.038, respectively). No difference was observed among the groups in terms of other parameters (all p>0.05).

Table 2 shows the ONH-BFA values measured by OCTA and the RNFL thickness values in all quadrant scans.

**Table 2:** Comparison of optic nerve head-blood flow area and retinal nerve fiber layer sectors among the three study groups by OCTA

	Group 1 (Mild-moderate FMS) (n=47)	Group 2 (Severe FMS) (n=38)	Group 3 (Control group) (n=38)	p-value
ONH-BFA (mm^2^)	1.61 ± 0.08	1.63 ± 0.09	1.49 ± 0.10^a,c^	0.001^*^
RNFL avr (µm)	103.21 ± 10.66^f^	101.18 ± 6.03	106.51 ± 8.88^d^	0.020^*^
RNFL sup-t (µm)	139.02 ± 10.03	135.60 ± 18.29	142.46 ± 14.32	0.102
RNFL sup-n (µm)	110.44 ± 11.07	107.73 ± 21.57	113.76 ± 17.07	0.271
RNFL inf-t (µm)	142.15 ± 17.79^f^	134.36 ± 12.19	144.70 ± 16.25^d^	0.010^*^
RNFL inf-n (µm)	117.94 ± 20.53^f^	109.47 ± 16.03	118.63 ± 19.01^d^	0.045^*^
RNFL n-sup (µm)	84.34 ± 13.09	85.26 ± 13.11	91.08 ± 12.11^b,d^	0.031^*^
RNFL n-inf (µm)	79.65 ± 11.19	77.26 ± 11.20	80.02 ± 10.26	0.468
RNFL t-sup (µm)	84.57 ± 11.99	82.42 ± 8.65	86.31 ± 9.94	0.224
RNFL t-inf (µm)	70.89 ± 8.48	69.76 ± 9.47	72.70 ± 9.58	0.335

*One-way ANOVA; Kruskal-Wallis H test; Post hoc test= Tukey HSD*.

* *p*≤0.05, The difference between the three groups was statistically significant

ONH-BFA= Optic nerve head-blood flow area; RNFL= Retinal nerve fiber layer, avr= average; sup-t= superotemporal sector; sup-n= superonasal sector; inf-t= inferotemporal sector; inf-n= inferonasal sector; n-sup= nasal-superior sector; n-inf= nasal-inferior sector; t-sup= temporal-superior sector; t-inf= temporal-inferior sector.

a p<0.01 between Groups 3 and 1,

b p<0.05 between Groups 3 and 1,

c p<0.01 between Groups 3 and 2,

d p<0.05 between Groups 3 and 2,

e p<0.01 between Groups 1 and 2,

f p<0.05 between Groups 1 and 2.

Regarding the association between the measured OCTA values and FMS disease severity, a weak positive correlation was determined between the values [ONH-BFA (r=0.354, p<0.001), average RNFL (r=0.199, p=0.027), inferotemporal RNFL (r=0.265, p=0.003), and inferonasal RNFL (r=0.217, p=0.016)] and the severity of the disease (Table 3).

**Table 3:** The correlation between FMS severity determined according to FIQ scores in patients and measured OCTA values

	r value	p-value
ONH-BFA (mm^2^)	0.354^**^	<0.001
RNFL avr (µm)	0.199^*^	0.027
RNFL sup-t (µm)	0.012	0.897
RNFL sup-n (µm)	0.017	0.853
RNFL inf-t (µm)	0.265^**^	0.003
RNFL inf-n (µm)	0.217^*^	0.016
RNFL n-sup (µm)	0.100	0.270
RNFL n-inf (µm)	0.037	0.682
RNFL t-sup (µm)	0.140	0.121
RNFL t-inf (µm)	0.025	0.785

*Spearman’s rho correlation analysis; r*= correlation cofficient.

* = Correlation is significant at the 0.05 level (2-tailed).

* ^*^= Correlation is significant at the 0.01 level (2-tailed).

* *p*≤0.05, The difference between the three groups was statistically significant

ONH-BFA= Optic nerve head-blood flow area; RNFL= Retinal nerve fiber layer; avr= average; sup-t= superotemporal sector; sup-n= superonasal sector; inf-t= inferotemporal sector; inf-n= inferonasal sector; n-sup= nasal-superior sector; n-inf= nasal-inferior sector; t-sup= temporal-superior sector; t-inf= temporal-inferior sector

**Figure 1:**
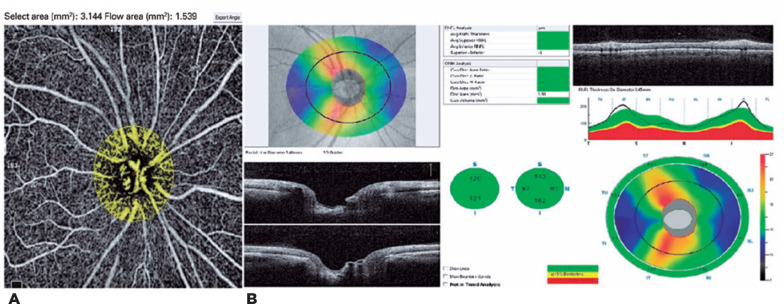
Optic nerve head-blood flow area and retinal nerve fiber layer, which were evaluated by OCTA in the mild-moderate FMS group. A) Optic nerve head-blood flow area (ONH-BFA). B) Retinal nerve fiber layer (RNFL).

**Figure 2:**
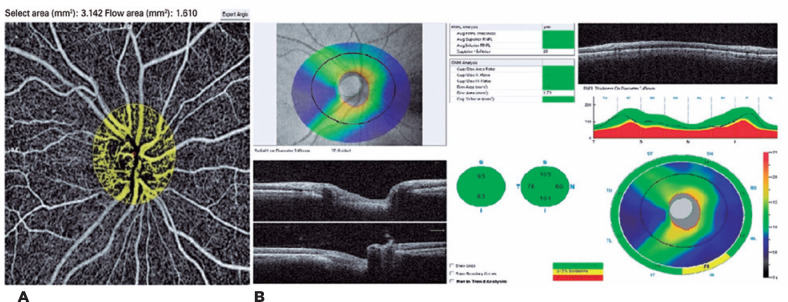
Optic nerve head-blood flow area and retinal nerve fiber layer, which were evaluated in the severe FMS group. A) Optic nerve head-blood flow area (ONH-BFA). B) Retinal nerve fiber layer (RNFL).

**Figure 3:**
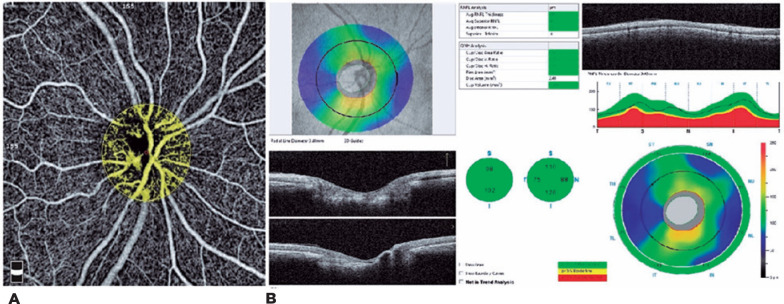
Optic nerve head-blood flow area and retinal nerve fiber layer, which were evaluated in the control group. A) Optic nerve head-blood flow area (ONH-BFA). B) Retinal nerve fiber layer (RNFL).

The images of ONH-BFA and RNFL thickness measurements of the three study groups are shown in figures 1-3.

## DISCUSSION

To the best of our knowledge, this is the first study to evaluate the quantitative changes in optic nerve head blood flow and RNFL thickness in patients with FMS by OCTA. Patients with both moderate and severe FMS showed greater ONH-BFA values than healthy controls, whereas the average RNFL thickness and several regional thickness values (inferotemporal, inferonasal, and nasal-superior quadrants) were reduced in proportion to disease severity. These findings confirm the involvement of the optic nerve in FMS and indicate that OCTA can provide objective noninvasive measures for the diagnosis and assessment of disease progression.

Several studies have suggested that optic nerve head shrinkage as measured by OCT can be used as a diagnostic marker of axonal loss in neurodegenerative disorders such as multiple sclerosis (MS), Parkinson’s disease, and Alzheimer’s disease^(10,11,17)^. In addition, although FMS is not a typical neurodegenerative disorder, there are several shared symptoms^(18)^. In fact, as in MS, a reduced RNFL thickness in the optic nerve head as measured by OCT was associated with the presence and severity of FMS. Similar to the findings of Garcia-Martin et al.^(18)^, thinning of inferotemporal fibers was also detected in our study in addition to reductions in average, inferonasal, and inferotemporal RNFL thicknesses. Moreover, these changes were indicative of FMS severity, underscoring the importance of RNFL thickness evaluation, especially of the temporal quadrant, in FMS (although thinning of the nasal quadrant fibers may also be indicative of FMS and should not be overlooked).

Although the mechanisms underlying FMS-associated neurodegeneration are uncertain, there is compelling evidence indicating the involvement of hypoperfusion and associated impairment of axon conduction. One study conducted using the calorimetric method found reduced hemoglobin levels in the optic disc and neuroretinal rim compared to those in a healthy group^(19)^. Similarly, Ulusoy et al. found reduced ocular choroidal flow in patients with FMS as evidenced by OCT measurement of choroidal thickness, which they attributed to autonomic system activation secondary to the cardiovascular response^(20)^. However, choroidal vascular structures were evaluated only using layer analysis due to the lack of measurement technology, and autonomic regulation is quite limited in this region. In contrast, we used OCT with split-spectrum angiography to analyze the vascular plexus of the retina and the optic nerve head by measuring the intravascular distribution of erythrocytes without the use of tracers or radiation^(21,22)^. These OCTA measures disclosed greater ONH-BFA values in patients with FMS, in contrast to those reported by several previous studies, as well as a weak correlation between ONH-BFA and increased disease severity.

Changes in catecholamine metabolism and dysfunction of the autonomic nervous system are also responsible for the etiopathogenesis of FMS. Catecholamines act as neurotransmitters in pathways that initiate and inhibit pain. It is believed that changes in catecholamine metabolism in FMS may cause clinical symptoms by both causing changes in pain sensation and impairment in autonomic dysfunction^(23,24)^. Several studies have reported reduced urinary catecholamine levels in patients with FMS (although contradictory results have also been reported), which may reflect the autonomic dysfunction in target organs^(25,26)^. We suggest that this enhanced ONH-BFA results from the dysfunction of the autonomic nervous system. Normally, the local autoregulation system of the eye ensures that blood flow remains constant and is not affected by systemic pressure, IOP, and the autonomic nervous system in vascular structures of the retina and optic nerve head^(27,28)^. Therefore, the increase in optic nerve head blood flow as shown by OCTA in both mild-moderate and severe FMS groups may be an indicator of vasodilatation caused by reduced catecholamine levels in the systemic circulation stemming from the autonomic dysfunction.

This study has several limitations. First, catecholamine levels in blood and/or urine were not measured as their potential importance was unforeseen. Determining the levels of urinary catecholamines may provide valuable information about sympathetic system dysfunction in non-muscle tissues of patients with FMS. Second, we did not explore the alterations in proinflammatory cytokines and chemokines, which have also been implicated in FMS pathogenesis^(29,30)^. Third, these findings apply only to female patients, although FMS is also present in male patients. Longer recruitment periods are required to confirm these OCTA findings in male subjects.

In conclusion, FMS has heterogeneous effects on neural and vascular structures of the eye that may be directly related to disease pathogenesis. OCT and OCTA examinations allow objective evaluation of neural and vascular structures of the eye and thus may be critical modalities for FMS diagnosis, monitoring of disease progression, evaluation of treatment response, and preclinical investigations on FMS pathophysiology.
